# Ethnomedicinal study of plants used in villages around Kimboza forest reserve in Morogoro, Tanzania

**DOI:** 10.1186/1746-4269-8-1

**Published:** 2012-01-06

**Authors:** Ezekiel Amri, Daniel P Kisangau

**Affiliations:** 1 Department of Science and Laboratory Technology, Dar es Salaam Institute of Technology, P. O. Box 2958, Dar es Salaam, Tanzania; 2 Department of Biological Sciences, South Eastern University College (A Constituent College of the University of Nairobi) P.O Box 170-90200, Kitui, Kenya

**Keywords:** Ethnobotany, Medicinal plants, Kimboza forest, Conservation

## Abstract

**Background:**

An ethnomedicinal study was conducted to document medicinal plants used in the treatment of ailments in villages surrounding Kimboza forest reserve, a low land catchment forest with high number of endemic plant species.

**Methods:**

Ethnobotanical interviews on medicinal plants used to treat common illnesses were conducted with the traditional medical practitioners using open-ended semi -structured questionnaires. Diseases treated, methods of preparation, use and habitat of medicinal plants were recorded.

**Results:**

A total of 82 medicinal plant species belonging to 29 families were recorded during the study. The most commonly used plant families recorded were Fabaceae (29%), Euphorbiaceae (20%), Asteraceae and Moraceae (17% each) and Rubiaceae (15%) in that order. The most frequently utilized medicinal plant parts were leaves (41.3%), followed by roots (29.0%), bark (21.7%), seeds (5.31%), and fruits (2.6%). The study revealed that stomach ache was the condition treated with the highest percentage of medicinal plant species (15%), followed by hernia (13%), diarrhea (12), fever and wound (11% each), and coughs (10%). Majority of medicinal plant species (65.9%) were collected from the wild compared to only 26.7% from cultivated land.

**Conclusions:**

A rich diversity of medicinal plant species are used for treating different diseases in villages around Kimboza forest reserve, with the wild habitat being the most important reservoir for the majority of the plants. Awareness programmes on sustainable utilization and active involvement of community in conservation programmes are needed.

## Background

Kimboza forest reserve has 13 recorded endemic plant species making it the richest lowland forest in East Africa. The forest reserve has valuable contribution to biological and gene pool conservation, and together with other mountain ranges of Morogoro region form part of the Eastern Highlands of Tanzania with about 200 endemic plant species [1,2]. The uses of plants in the indigenous cultures particularly of developing countries, are numerous and diverse, forming an important socio-economic base including their use as medicine [3]. People generally depend on nearby forests for fuel wood, timber and medicine. Medicinal plants therefore have important contribution in the primary healthcare systems of local communities as the main source of medicines for the majority of the rural population [4,5].

The World Health Organisation (WHO) estimates that up to 80% of the world's population in developing countries depend on locally available plant resources for their primary healthcare, since western pharmaceuticals are often expensive, inaccessible or unsuitable [6]. Further, in this decade, the world is experiencing an increasing rate of resistance by pathogens to some of the synthetic drugs, as well as the struggle against some chronically complex and uncontrolled infections such as Cancer and HIV/AIDS. There is therefore need to study and validate ethnomedicines for wider acceptance, recognition and utilization by all stakeholders in the society. However, overtime, ethnomedicinal knowledge has been undermined by mortality of resource persons, extinction of plant resources, inadequate scientific research and poor documentation [7]. The aim of the present study was therefore to document ethnomedicinal information of plants used by indigenous people in villages surrounding Kimboza forest reserve. The generated information will be used in future to explore ways of sensitizing the community on the sustainable utilization of the forest resources so as to minimize their genetic loss.

## Methods

### Study area

**Figure 1:**
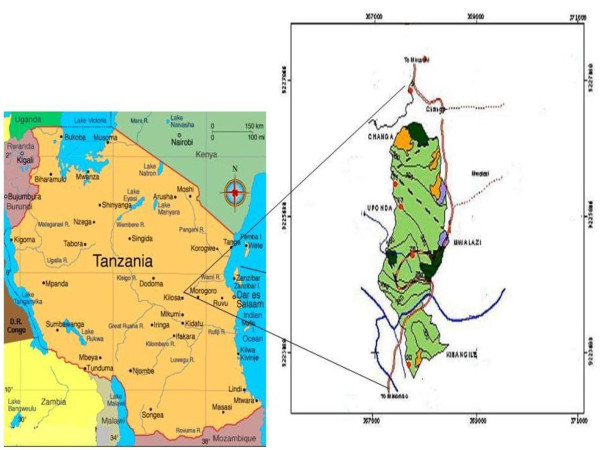
**Map showing location of the study area**.

The study area is about 60 km from Morogoro Municipal located between 06°59' - 7°02' S and 37°47' - 37°49'E. An ethnobotanical survey for medicinal plants was conducted in the following villages: Changa, Kibangile, Mwarazi and Uponda which surround Kimboza forest reserve (Figure 1). The communities adjacent to the forest have access right over the forest as stipulated in the village forest management plan by-laws.

### Data collection

Ethnobotanical surveys were carried out to obtain information on medicinal plants traditionally used to treat various ailments in the study area. With the help of local administrative officers, Participatory Rural Appraisal (PRA) was employed to identify key respondents [8]. Ethnobotanical data was collected through individual and Focus Group Discussion (FGD) interviews using semi-structured open-ended questionnaires. Interviews were conducted in Swahili or the local Kiluguru language. Field excursions were also conducted with the assistance of key respondents. Information regarding the local names of the plant species, medicinal uses, parts used, methods of preparation, and administration route were documented. The methods used in harvesting the plant materials from the wild were also recorded. Plant specimens were prepared, identified and voucher specimens deposited in the University of Dar es Salaam herbarium for future reference. Descriptive statistics were used to analyze the ethnomedicinal data collected.

## Results and discussion

**Figure 2:**
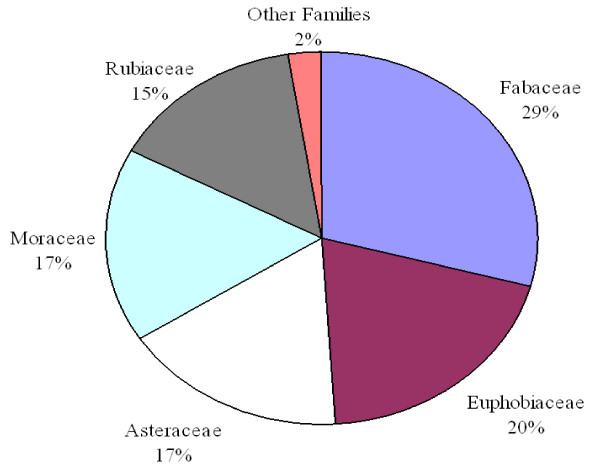
**Percentage distribution in families for medicinal plants used treat different health problems**.

A total of 22 traditional medical practitioners were interviewed, with their ages ranging between 28 to 70 years, and 60% of them being older than 50 years. Out of the 22 informants interviewed, 18 of them were men and only four were women. A total of 82 medicinal plant species in 29 families were recorded as being used to treat 41 different types of diseases as shown in Table 1. The family Fabaceae had the highest proportion of medicinal plants used (29%), followed by Euphorbiaceae (20%), Asteraceae and Moraceae (17% each), and Rubiaceae (15%) in that order (Figure 2). Each of all other families had less than six plant species associated with the treatment of the diseases documented in Table 1.

**Table 1:** Medicinal plants used for treating human diseases in villages around Kimboza forest reserve

Family/Botanical name	Local name	Habitat/Source	Part used	Health problems cured	Methods of preparation	Administration route	**Voucher No**.
**Acanthaceae**							
*Justicia heterocarpa *L.	Mwidu	Wild or Cultivated	Roots, Leaves	Snake bite	Crushed or pounded	Oral	EA031
**Alliaceae**							
*Allium ascalonium *L.	Kitunguu saumu	Cultivated	Roots, Leaves	Antifungal, Child fever	Decoction	Oral	EA040
*Allium sativum *L.	Kitunguu maji	Cultivated	Leaves	Fever, Cough, Asthma, Malaria, Hypertension	Concoction	Oral	EA017
**Anacardiaceae**							
*Sorindeia madagascariensis *DC.	*Mpilipili*	Wild	Leaves	Wound	Crushed or pounded	Topical	EA021
*Mangifera indica *L.	Mwembe	Cultivated	Leaves, Bark, Roots	Malaria,Diarrhea, Hypertension, Asthma, Cough	Decoction	Oral	EA025
**Annonaceae**							
*Annona senegalensis *Pers.	Mtopetope	Wild	Roots Leaves	Eye ache, Wound	Crushed or pounded	Topical	EA035
							
**Apocynaceae**							
*Landolphia buchananii *P.Beauv.	Luziwana	Wild	Roots	Stomach ache, Diarrhea, Hernia	Decoction	Oral	EA022
**Asteraceae**							
*Vernonia iodocalyx *O. Hoffm.	Kitugutu	Wild	Bark leaves	Stomach ache, Diarrhea, Head ache	Crushed or pounded	Oral	EA010
*Helichrysum schimperi *(Sch. Bip. ex A. Rich.) Moeser	Lweza	Wild	Roots	Stomach ache, Diarrhea,	Decoction	Oral	EA013
*Vernonia hymenolepis *A. Rich.	Msungu	Wild	Roots, Leaves	Fever, Diarrhea, Hernia, Spleen enlargement.	Decoction	Oral	EA006
*Crassocephalum* *Montuosum *(S.Moore) Milne-Redh.	Nyaluganza	Wild	Bark	Ear ache, Head ache, burn	Crushed or pounded	Oral	EA004
*Bidens pilosa *L.	Nyaweza	Wild, Cultivated	Bark	Wound, Spleen enlargement	Decoction	Topical	EA003
*Sonchus pinnatifidus *L.	Sungasunga	Wild	Roots, Leaves	Stomachache, Headache.	Decoction	Oral	EA032
*Helianthus annus *L.	Ufuta	Cultivated	Leaves	Chest pain, Asthma	Concoction	Oral	EA002
**Asphodelaceae**							
*Aloe vera *(L.) Burm.f.	Mlovera	Cultivated	Leaves	Skin diseases, Wounds	Crushed or pounded	Topical	EA041
							
**Bignoniaceae**							
*Kigelia africana *(Lam.) Benth.	*Mwegea*	Wild	Bark, Leaves, Fruits	Skin diseases, Venereal diseases, Fever,	Crushed or pounded	Oral	EA026
**Bombacaceae**							
*Adansonia digitata *L.	Mbuyu	Wild	Roots	Hernia	Decoction	Oral	EA018
*Bombax rhodognaphalon *L.	Msufipori	Wild	Leaves	Stomach ache	Decoction	Topical	EA024
**Caricaceae**							
*Carica papaya *L.	Mpapai	Cultivated	Roots, Leaves	Tooth ache, Stomach-ache.	Decoction	Oral	EA015
**Combretaceae**							
*Cobretum molle *R.Br. ex G.Don.	Mlamweusi	Wild	Roots, Leaves	Wounds, Cough, Malaria	Decoction	Oral	EA012
*Terminalia sericea *L.	Mpululu	Wild	Leaves, Roots	Antifungal treatment	Crushed and pounded	Topical	EA053
**Cucurbitaceae**							
*Momordica foetida *L.	Huluhundu	Cultivated	Leaves	Malaria, Ear ache, Head ache,	Decoction	Oral	EA048
*Cucurbita moschata *Duchesne.	Maboga	Cultivated	Roots	Expulsion of placenta	Infusion	Oral	EA042
**Euphorbiaceae**							
*Acalypha fruticosa *Forssk.	Kifulwe	Wild	Leaves	Diarrhea	Decoction	Oral	EA028
*Jatropha curcas *L.	Mbono	Wild	Leaves Seeds	Wound, Scabies	Crushed and pounded	Topical	EA034
*Fluggea virosa *Willd.	Mkalananga	Wild	Leaves	Stomach ache, Diarrhea, Hernia, Spleen enlargement	Infusion	Oral	EA029
*Manihot esculenta *Crantz.	Mhogo	Cultivated	Leaves	Stomach ache	Infusion	Oral	EA043
*Suregada zanzibariensis *Roxb. ex Rottler.	Mndimu pori	Wild	Roots	Malaria, Fever	Decoction	Oral	EA050
*Ricinus communis *L.	Mnyonyo	Wild	Leaves	Rheumatism, Wound	Crushed and pounded	Topical	EA055
*Bridelia micrantha *(Hochst.) Baill.	Msumba	Wild	Bark Leaves	Rheumatism, Hernia, Stomach ache, Spleen enlargement, Skin eruption, Insecticide	Decoction	Oral	EA036
*Euphorbia nyikae *Pax ex Engl.	Mngwede	Wild	Leaves	Wound	Crushed and pounded	Topical	EA044
**Fabaceae**							
*Cassia mimosoides *L.	Lusangalala	Wild	Roots Bark	Mental illness, Asthma, Severe cough	Decoction	Oral	EA056
*Senna petersiana *(Bolle) Lock.	Mkunde	Wild	Roots Leaves	Skin diseases, Inflammation Backache, Stomach ache, Skin eruption	Infusion	Oral	EA054
*Senna hirsuta *(L.) Irwin & Barneby.	Mwambalasimba	Wild	Leaves	Pneumonia, Hernia, Stomach ache, Head ache	Decoction	Oral	EA052
*Brachystegia spiciformis *Benth.	Mzinghawa nyika	Wild	Roots	Ear ache, Child fever.	Infusion	Oral	EA061
*Albizia versicola *Welw. ex Oliv.	Mkenge maji	Wild	Roots Bark	Skin diseases, Boils	Crushed or pounded	Topical	EA057
*Albizia petersiana *Oliv.	Mkenge poli	Wild	Leaves Bark	Skin diseases	Crushed or pounded	Topical	EA063
*Mucuna pruriens *(L.) DC	Bumu	Wild	Roots	Male fertility	Infusion	Oral	EA066
*Tephrosia vogelii *Hook.f.	Kitupa	Wild	Bark	Insecticide	Crushed or pounded	Topical	EA068
*Abrus precatorius *L.	Lufambo	Wild	Roots	Eye inflammation, Diarrhea, Women fertility	Decoction	Oral	EA078
*Cajanus cajan *(L.) Millsp.	Mbaazi	Cultivated	Leaves	Diarrhea.	Crushed or pounded	Oral	EA071
*Vigna unguiculata *(L.) Walp.	Mkunde	Cultivated	Roots Leaves	Chest pain, Cough, Abscess, Hernia	Infusion	Oral	EA062
*Pterocarpus angolensis *DC.	Mninga	Wild	Bark	Hernia	Decoction	Oral	EA064
**Lamiaceae**							
*Satureja biflora *(Buch.- Ham.ex D.Don) Briq.	Luparalwa mlungu	Wild	Leaves	Mental illness	Infusion	Oral	EA011
*Ocimum suave *Willd.	Mnung'ha	Wild	Bark	Malaria, Stomach ache,	Decoction	Oral	EA001
**Lauraceae**							
*Ocotea usambarensis *Engl.	Mseli	Wild	Roots Bark	Stomach ache, Fever, Malaria, Hernia, Sprit	Infusion	Oral	EA009
**Malvaceae**							
*Hibiscus surattensis *L.	Lumotomoto	Wild Cultivated	Leaves	Wound, Abscess, Gonorrhea	Crushed or pounded	Topical	EA059
*Hibiscus fuscus *Garcke	Luswagamene	Wild	Roots	Rheumatism, Mental illness.	Concoction	Oral	EA043
**Melastomataceae**							
*Dissotis rotundifolia *(Sm.) Triana.	Kinzasu	Wild	Roots, Leaves	Wound, Asthma, Boil, Abscess Diarrhea, Gonorrhea	Crushed or pounded; Decoction	Topical; Oral	EA039
**Meliaceae**							
*Khaya anthotheca *(Welw.) C. DC	Mkangazi	Wild	Bark Leaves	Malaria, Bilharzias, Gonorrhea	Concoction	Oral	EA067
*Azadirachta indica *A. Juss.	Mwarobaini	Cultivated	Leaves, Bark, Seeds	Head ache, Back ache, Malaria, Fever, Stomach-ache, Insecticide	Decoction	Oral	EA080
*Cedrella odorata *L.	Mwerezi	Wild	Leaves Bark	Menstrual cycle and Women fertility	Infusion	Oral	EA079
**Moraceae**							
*Ficus altissima *Blume.	Mvira	Wild	Bark Leaves	Diarrhoea, Stomach-ache,	Concoction	Oral	EA077
*Ficus asperifolia *Hook. ex Steud.	Mkoya	Wild	Roots Leaves	Wounds	Crushed or pounded	Topical	EA075
*Ficus exasperata *Valh	Msasa	Wild	Roots Leaves Bark	Hypertension, Scabies, Stomach disorders, Gonorrhoea, Diarrhea	Decoction	Oral	EA073
*Ficus stuhlmanii *Warb.	Foza/Mtamba	Wild	Bark	Stomach tumor	Infusion	Oral	EA069
*Ficus sycomorus *L.	Mkuyu	Wild	Bark	Menstrual cycle, Women fertility	Infusion	Oral	EA067
*Milicia excelsa *(Welw.) C.C Berg.	Mvule	Wild	Roots	Wound	Crushed or pounded	Topical	EA082
*Antiaris toxicaria *Lesch.	Mbila	Wild	Leaves Bark	Skin diseases, Insecticide.	Crushed or pounded	Topical	EA088
**Moringaceae**							
*Moringa oleifera *Lam.	Mlonge	Cultivated	Leaves Bark Seeds Roots	Skin diseases, headache, [Detoxification, Rheumatism, inflammation	Decoction or Infusion	Oral	EA097
**Myrtaceae**							
*Eucalyptus maidenii *Labill.	Mmaidini	Wild Cultivated	Bark	Chest pain, Cough	Decoction	Oral	EA087
*Psidium guajava *L.	Mpera	Cultivated	Leaves	Diarrhea, Stomach ache	Infusion	Oral	EA085
**Poaceae**							
*Zea mays *L.	Mahindi	Cultivated	Roots	Bedwetting	Decoction	Oral	EA091
*Saccharum officinarum *L.	Muwa	Cultivated	Roots	Bilharzias, Detoxifying kidneys	Decoction	Oral	EA101
**Podocarpaceae**							
*Podocarpus latifolius *(Thunb.) R.Br. ex Mirb.	Mnyanziri	Wild	Roots	Hernia	Decoction	Oral	EA99
**Rosaceae**							
*Rubus pinnatus *Willd	Lufifi	Wild	Leaves	Menstrual cycle,	Infusion	Oral	EA093
*Prunus americana *Marshall.	Mpisi	Wild	Bark	Stomach ache	Crushing and water	Oral	EA090
**Rubiaceae**							
*Multidentia fanshwei *(Tennant) Bridson.	Degedege	Wild	Roots	Child fever	Decoction	Oral	EA095
*Rytigynia lichenixenos *(K.Schum.) Robyns.	Mhambalamaziwa	Wild	Seeds Roots	Hernia	Decoction	Oral	EA092
*Vangueria infausta *Burch.	Mviru	Wild	Seeds	Pneumonia, Menstrual cycle,	Infusion	Oral	EA084
*Rytigynia uhligii *(K.Schum. & K.Krause) Verdc.	Msanangare	Wild	Seeds	Hernia	Decoction	Oral	EA099
*Chassalia parvifolia *K. Schum.	Mguhu	Wild	Bark	Hernia, Chest pain, Malaria,	Concoction	Oral	EA102
*Catunaregum spinosa *(Thunb.)	*Mtutuma*	Wild	Leaves	Skin diseases	Crushed or pounded	Topic	EA104
**Rutaceae**							
*Citrus limon *(L.) Burm.f.	Mlimau	Cultivated	Roots	Stomach ache	Decoction	Oral	EA103
*Zanthoxylum deremense* (Engl.)	Mlungulungu	Wild	Fruits	Stomach ache, Child fever	Decoction	Oral	EA100
*Citrus aurantifolia *(Christm.) Swingle.	Mndimu	Cultivated	Roots	Gonorrheal, Diarrhea,	Decoction	Oral	EA117
**Solanaceae**							
*Lycopersicum esculentum *Mill.	Mnyanya	Cultivated	Roots, Leaves	Stomach ache	Concoction	Oral	EA105
*Solanum incanum *L.	Mtula	Wild Cultivated	Leaves	Cough, Vomit.	Concoction	Oral	EA107
*Capsicum frutescens *L.	Pilipililukwale	Cultivated	Roots, Bark	Wound	Crushed or pounded	Topical	EA110
*Nicotiana tabacum *L.	Tumbaku	Cultivated	Fruits	Hernia	Decoction	Oral	EA106
**Zingiberaceae**							
*Zingiber officinale *Roscoe.	Tangawizi	Cultivated	Roots	Cough	Decoction	Oral	EA111

**Figure 3:**
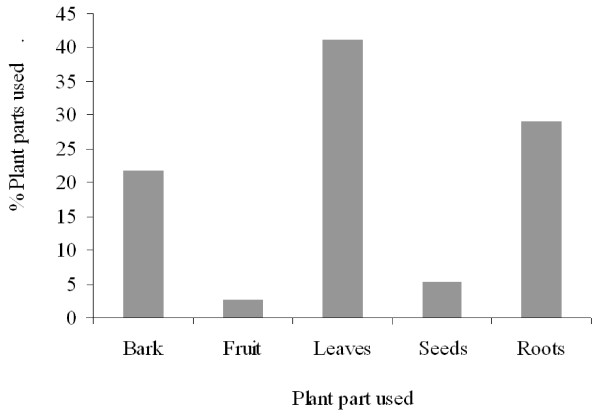
**Plant parts used for medicinal purposes and percentage of total medicinal species**.

At least 65.9% of all medicinal plants recorded were collected from the wild while only 26.7% were collected from cultivated land. The most frequently utilized medicinal plant parts were leaves (41.3%), followed by roots (29.0%), bark (21.7%), seeds (5.3%), and fruits (2.6%) as shown in Figure 3. Roots were mostly used in the treatment of stomachache, diarrhea and inflammatory diseases, while leaves were mostly used in the treatment of malaria, skin diseases and children's diseases.

**Figure 4:**
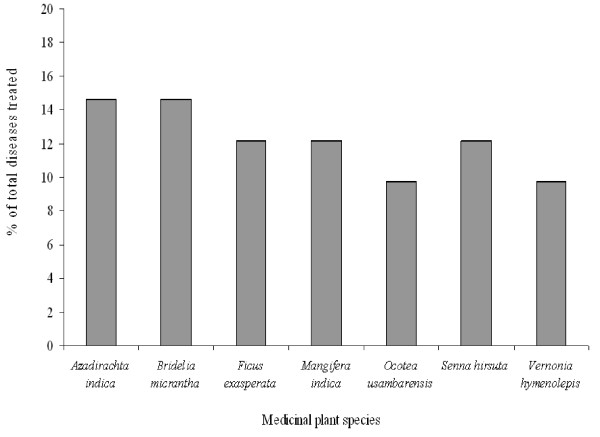
**Medicinal plant species with highest percentage of total diseases treated**.

**Figure 5:**
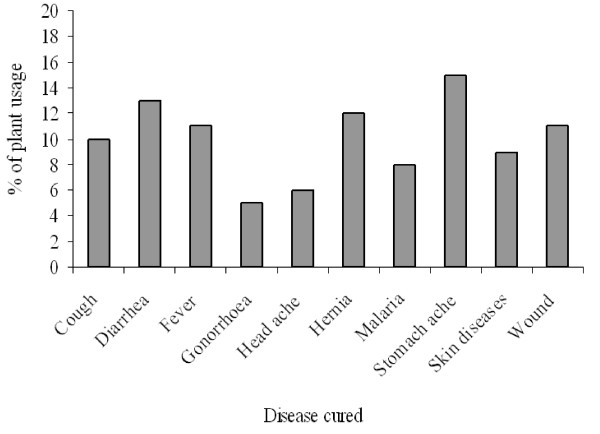
**Frequency of medicinal plants uses to cure diseases**.

Most of the plant species were used to treat one disease, while some were used to treat two or more diseases. The plant species used to treat the highest percentage of diseases were Azadirachta *indica *A. Juss. and *Bridelia micrantha *(Hochst) Baill. each reported to treat 14.6% of the diseases. *Ficus exasperate *Valh.*, Mangifera indica *L. and *Senna hirsuta *(L.) Irwin & Barneby. were each reported to treat 12.2% of the diseases. The third category of highly used plant species were *Ocotea usambarensis *Engl. and *Vernonia hymenolopis *A. Rich. each reported to treat 9.8% of the diseases (Figure 4). In terms of frequency of medicinal plant uses, the highest percentage of plant species (15%) was used to treat stomachache, followed by diarrhea (13%) and hernia (12%), fever and wound (11% each) and cough (10%). Other diseases were treated with less than 10% of the medicinal plants recorded (Figure 5).

**Figure 6:**
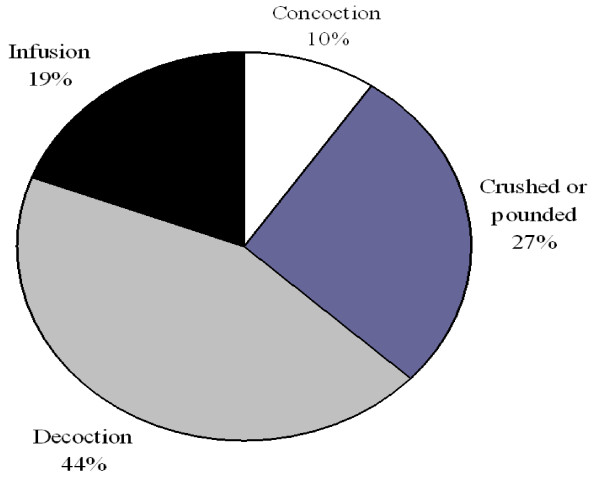
**Medicinal plants preparation methods**.

Majority of the plant remedies were prepared by boiling (44%), then by crushing or pounding (27%), or soaking in cold water (19%) and concoction 10% (Figure 6). Some specific herbal preparations were taken by mixing with food, honey or drunk together with coffee prepared from leaves of the coffee plant. Most medicinal plant preparations were taken orally (75.6%), while 24.4% were administered topically for diseases such as skin infections and wounds. It was reported that different dosages were given to similar disease conditions. Administration dosage was by estimation and the amount of each dosage depended on the age of the patient and severity of the condition being treated.

The study revealed a rich diversity of medicinal plants used to treat various disease conditions in the villages around Kimboza forest reserve. It further revealed a rich ethnobotanical knowledge amongst the residents of the neighbourhood of Kimboza Forest reserve. The families Fabaceae, Euphorbiaceae, Asteraceae, Moraceae and Rubiaceae which were the most dominant in this study are consistently recorded in other ethnomedicinal studies [9-12], and could be attributed to their wide range of bioactive ingredients [13,14].

The fact that majority of the plant species recorded were sourced from the wild and only a few were cultivated may imply that many indigenous plant species may be difficult to propagate. This therefore calls for the need to train the herbal practitioners on the appropriate propagation techniques of these plant species for sustainable utilization. According to Edwards [15], about two-thirds of 50, 000 medicinal plants in use worldwide are still harvested from the natural habitat and about one fifth of them are now endangered. The increased percentage of species obtained from the wild has a direct effect on the availability of these resources and is likely to contribute to their vulnerability to being over-exploited.

The finding that majority of the informants interviewed were aged above 50 years augments Kisangau *et al. *[16]. This implies that the elderly people are the main custodians of traditional knowledge, and this poses a serious challenge of the knowledge gap between the elderly and the young generation if framework to ensure apprenticeship is not put in place.

Some herbal practitioners reported that there was a potential to domesticate medicinal plants as some of them were already being planted on farmlands. Kisangau *et al. *[17] support the observation that only a few herbal practitioners were involved in cultivation of medicinal plants and most of them were gathered from the wild. The unabated over collection of the medicinal plants from the wild is a major threat to their existence and raises serious concern for their conservation. In the present study, the few plant species that were found to be cultivated on farmlands included *Senna petersiana *(Bolle) Lock., *Azadirachta indica *A. Juss., *Khaya anthotheca *(Welw.) C. DC. and *Moringa oleifera *Lam. However, unavailability of planting material and lack of appropriate propagation techniques were noted to be the major constraints to exploiting the potential for medicinal plant domestication. On the other hand, species like *Carica papaya L*., *Vernonia iodocalyx *O. Hoffm., *Helichrysum schimperi *(Sch. Bip. ex A. Rich) Moeser and *Citrus aurantifolia *(Christm) Swingle. could easily be conserved by planting them in home gardens.

It was found that the most commonly harvested plant parts were leaves followed by roots. These results are contrary with the findings of Rukia [18] who reported that roots were the most commonly harvested plant parts followed by leaves in Udzungwa Mountains Forests in Tanzania.

Some methods of harvesting medicinal plants like root excavation and bark striping can be very devastating and a big threat to the plant survival. The high utilisation of roots has also been reported as putting many plant species at a risk of extinction because of the damages inflicted on them in the course of uprooting them [19,20]. Bark striping is also an equally harmful harvesting method as reported for *Prunus africana *and other medicinal plants in Cameroon [7]. In Namibia just like in other countries, harvesting of roots and barks was found to be common, destructive and unsustainable [21].

The fact that the most frequently utilised plant parts were leaves is a more sustainable practice as opposed to where roots and/or the bark are used. The prevalence in the use of leaves for preparation of traditional herbal remedies has been reported in other studies too [9,22-26]. This practice helps to reduce the rate of threat on plant species and enhances the sustainable management of plants, as long as only an appreciable amount of leaves is harvested [27]. Leaves of plants have been reported to accumulate inulins, tannins and other alkaloids [28], which may be responsible for their various medicinal properties, hence explaining their wide use.

## Conclusion

The results of the study revealed that there is rich diversity of medicinal plants used to treat various ailments in the neighbourhood of Kimboza forest reserve. Herbal practitioners and the local community in the study area should be educated on sustainable methods of harvesting medicinal plants without compromising their availability for future use. It is also imperative to train the community on the proper propagation techniques in order to encourage the domestication of valuable and threatened medicinal plants. The domestication of medicinal plants will create new opportunities for the local people such as provision of an alternative income and could help reduce the pressure on the wild population. Successful conservation strategies should be developed and priority given to sustainable harvesting of the plants.

## Competing interests

The authors declare that they have no competing interest, and share the aspirations of the local people in the villages around Kimboza forest reserve to conserve medicinal plants for future generations.

## Authors' contributions

EA identified the research area and title, involved in field data collection, carried out statistical analysis and drafted the manuscript. DPK participated in refining data analysis and drafting as well as enrichment of the manuscript. All authors read, revised and approved the final manuscript.
